# A conducive clinical learning environment in primary care as a wicked problem: A scoping review of enablers, obstacles, and strategies

**DOI:** 10.4102/phcfm.v18i1.5246

**Published:** 2026-08-06

**Authors:** Owen Eales, Hanneke Brits, Irene Lubbe, Maureen Moyo-Chilufya, Marietjie van Rooyen

**Affiliations:** 1 Department of Family Medicine, Faculty of Health Sciences, University of Pretoria, Pretoria, South Africa; 2 Department of Family Medicine, Faculty of Health Sciences, University of the Free State, Bloemfontein, South Africa; 3 Department of Future Learning and Development, University of Canterbury, Christchurch, New Zealand; 4 School of Health Systems and Public Health, Faculty of Health Sciences, University of Pretoria, Pretoria, South Africa; 5 Department of Health and Rehabilitation Sciences, Division of Physiotherapy, Stellenbosch University, Cape Town, South Africa

**Keywords:** clinical learning environment, primary care, decentralised training, medical students, wicked problem, faculty development, educational policy

## Abstract

**Background:**

The clinical learning environment (CLE) in primary care is increasingly recognised as critical to undergraduate medical education. However, its design and implementation are shaped by multiple interdependent factors, such as mentorship, autonomy, relationships and logistics, which make it a complex challenge.

**Aim:**

This study aimed to map the literature on the contributing factors to conducive CLE for medical students in primary care settings, identify enablers and obstacles and strategies for improvement.

**Setting:**

Primary care clinical learning environments, including community health centres, primary healthcare clinics, general practices, and district hospitals involved in undergraduate medical education.

**Methods:**

A scoping review was conducted using the Joanna Briggs Institute methodology and the PRISMA-ScR (Preferred Reporting Items for Systematic reviews and Meta-Analyses extension for Scoping Reviews) checklist. Four databases were searched with no language or date restrictions. Articles were screened independently by two reviewers. Data were charted using Gruppen’s conceptual framework and thematically synthesised.

**Results:**

A total of 34 articles were included. Common enablers included effective mentorship, student autonomy, positive interpersonal relationships, organisational alignment and supportive infrastructure. Obstacles included inadequate space, transport constraints, authoritarian teaching styles and a lack of student agency. Few articles proposed solutions, and many challenges were systemic and persistent. The CLE in primary care emerged as a *wicked problem* – a complex, context-sensitive issue involving multiple stakeholders without a definitive solution.

**Conclusion:**

A conducive CLE in primary care requires coordinated, multilevel strategies that address both human and structural domains. Isolated interventions are unlikely to produce lasting change.

**Contribution:**

This review highlights the CLE in primary care as a wicked problem and provides a framework for developing systemic, adaptive responses to support medical education in diverse contexts.

## Introduction

The clinical learning environment (CLE) is where students learn while interacting with patients.^1,2,3,4^ It represents the intersection of the educational context and the work environment, enabling students to actively participate in healthcare delivery rather than remaining passive observers. The CLE is where students learn patient communication and develop a ‘bedside manner’.^2,4,5^ The CLE has multiple impacts on students: a positive environment supports skills development, professional identity formation, and satisfaction, while an adverse environment hinders learning, reduces motivation, and impairs professional development.^6,7^ Evidence from a scoping review that focused on nursing students suggests that motivated clinical mentors are central to a conducive CLE, and they should be carefully selected and supported. Role clarification, dedicated time for teaching activities and professional development are foundational enablers. A supportive relationship between the student, the mentor and the clinical staff, as well as formal partnerships with educational institutions, contribute to a positive environment.^8,9^ The CLE in academic hospitals has been investigated extensively, but research about the CLE in primary care is limited.^2,4,8,9^ Primary care platforms create an authentic learning environment that equips students better for ‘real-world’ conditions that they face after graduation. Training in primary care fosters generalist skills, exposes students to the social determinants of health, and increases the capacity for universities to train more students.5 The World Health Organization (WHO) highlights that hospital-based CLEs are often disconnected from the social realities in which patients live and where they fall ill. Medical students trained only in academic hospitals are ill-prepared to serve in local communities. After graduation, they often move to larger urban areas or abroad to seek professional integration and apply their skills.^10^ Although related concepts such as Primary Health Care (PHC), decentralised training and rural education overlap with primary care training, they are not synonymous. Distinguishing these terms is essential to accurately interpret the literature on CLEs.

***Primary care*** is also understood as first-contact medicine that provides person-centred, continuous, comprehensive and coordinated care for undifferentiated health concerns across the lifespan, delivered in settings such as general practice, clinics and district hospitals.^10^ Frenk et al. advocated for a shift towards community-based primary care education in their landmark article on health professionals for a new century.^11^

***Primary Health Care*** is a more comprehensive concept and includes primary care, prevention, health promotion, and community participation to achieve universal health coverage. The Declaration of Alma-Ata in 1978 and the updated Declaration of Astana in 2018 affirmed PHC as the foundation of health systems worldwide and a key determinant of improved health outcomes.^12^

***Rural training platforms*** focus on training outside urban centres, are often resource-constrained and deal with staff shortages.^13^

***Decentralised training platforms*** focus on training activities conducted outside of major academic health centres, often in community clinics, rural hospitals, or other healthcare facilities.^14^

***Distributed training platforms*** include multiple locations, potentially including centralised and decentralised settings.^15^

***A conducive CLE*** is understood to facilitate learning, clinical participation and professional identity formation.^2^ This presents a complex, multifactorial challenge involving mentorship, autonomy, infrastructure, logistics, and institutional alignment. These components interact unpredictably, creating persistent tensions between educational goals and clinical service demands. Problems, such as this that are difficult to define, have no single solution and involve multiple stakeholders with conflicting interests, have been described in the literature as ‘wicked problems’.^16,17,18^

### Rationale for scoping review

Although reviews have been carried out on the CLE in primary care for nursing students^19^ and on the decentralised and rural training platforms,^20,21^ this review focuses on a conducive CLE for medical students. A scoping review is the best tool for a broad overview of the relevant concepts.

### Scoping review question

What is understood about the factors that contribute to a conducive CLE for medical students in primary care?

### Sub-questions

This overarching question will be addressed through the following sub-questions:

- What are the enablers and obstacles to a conducive CLE for medical students in primary care?
- What are successful strategies to improve the CLE for medical students in primary care?

## Research methods and design

The lead author, a qualified primary care family physician, coordinates medical student rotations in family medicine within primary care and therefore brings an embedded perspective on the CLE. This position provides contextual insight into the organisation of training in these settings.^22^ Reflexive awareness of this perspective was maintained during the review and synthesis.^23^ The multidisciplinary backgrounds of the co-authors (nursing, public health and higher education) contributed to a balanced interpretation of the findings.

### Protocol and registration

This scoping review was conducted in line with the Joanna Briggs Institute (JBI) methodology for scoping reviews and the framework of Arksey and O’Malley, with enhancements by Peters et al.^24^ Reporting followed the PRISMA-ScR checklist.^25^ The review protocol was registered on the Open Science Framework (https://doi.org/10.17605/OSF.IO/WV4MA) on 19 June 2025. This registration was completed *retrospectively*, after the initial database searches (09 March 2025–29 March 2025). The retrospective registration reflects the sequencing of project logistics rather than deviations from the protocol established before conducting the review.

### Search strategy

A three-step search strategy was employed, as recommended by the JBI. Firstly, a pilot search was conducted in PubMed Scopus, and EBSCOhost on 09 March 2025 to refine keywords and Boolean operators. Secondly, a comprehensive search was undertaken across PubMed, Scopus, Web of Science and EBSCOhost on 29 March 2025. No date or language restrictions were applied. The complete search strategy is provided in Online Appendix 1. Finally, the reference lists of key articles were screened manually for additional studies.^4,25^

Grey literature was excluded to ensure a reproducible search strategy across indexed databases. This may have resulted in the omission of relevant policy documents or implementation strategies and may introduce some publication bias.

### Eligibility criteria

Eligibility was defined using the Population-Concept-Context (PCC) framework (Table 1). The population of interest was undergraduate medical students including Bachelor of Medicine and Bachelor of Surgery (MBChB, MBBS, MBBCh, BMBS), Doctor of Medicine (MD), and Doctor of Osteopathic Medicine (DO) programmes. The concept was the CLE, defined as patient-facing settings where learning occurs through clinical interaction. The context was primary care, including clinics, community health centres, district hospitals and general practices. Studies conducted in district hospitals were included only when the educational activities described occurred within generalist or family medicine services exclusions applied to postgraduate students, other health professions, homeopathy, simulation-based training, lectures, outreach programmes and hospital-based learning environments. Interprofessional studies were included if data about medical students could be extracted. Only primary research articles were considered, and thus data synthesis and opinion articles were excluded.

**TABLE 1:** Population-concept-context framework.

PCC framework	Definition	Inclusions	Exclusions
Population: Undergraduate medical students	Students who study towards a medical degree to become a doctor	Undergraduate medical students This includes: MBChB (*Medicinae Baccalaureus Baccalaureus Chirurgiae*) MD (*Medicinae Doctor*) DO (*osteopathic medicine*) MBBCh (*Medicinae Baccalaureus et Baccalaureus Chirurgiae*) *BMBS (Bachelor of Medicine, Bachelor of Surgery)*	Postgraduate students, all other health sciences students, and homoeopathic students
Concepts: CLE	The clinical environment where students learn while they work	A clinical environment where students learn through direct interaction with patients and the healthcare team.	Learning that happens through didactic lectures, online learning, simulation training and non-clinical training
Context: Primary care	Essential medical care provided in communities close to where patients live	Clinics, community health centres, general practices, and district hospitals; first-contact medicine	Hospital-based care, school health programmes and community outreach programmes

Note: Detailed content and keyword search-strings can be found in Online Appendix 1.

CLE, clinical learning environment; PCC, population-concept-context.

### Selection of sources of evidence

**FIGURE 1:**
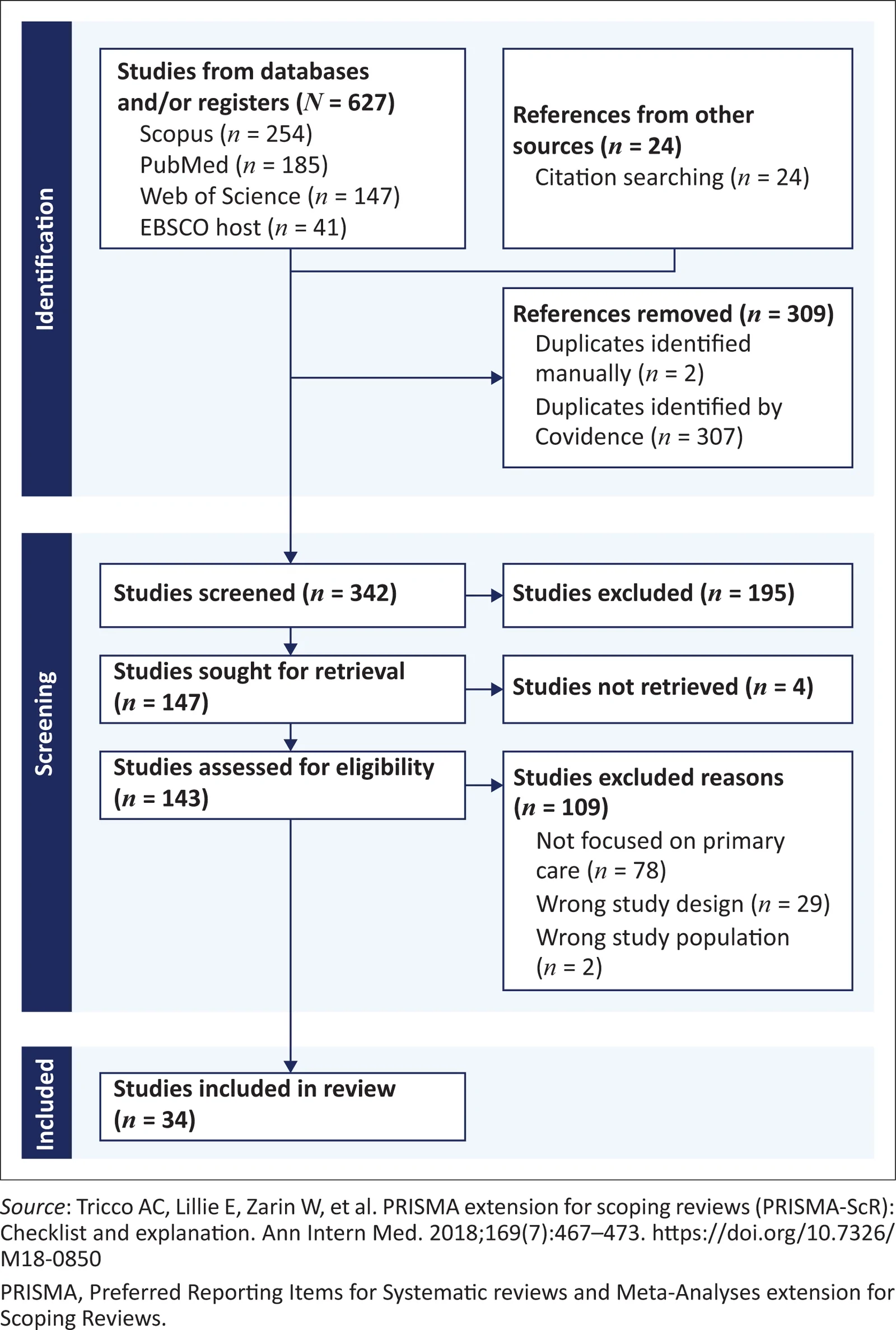
PRISMA flow chart.

After the search, all identified citations were imported into the Covidence software package, and duplicates were removed. The first stage involved screening titles and abstracts against the inclusion criteria by two reviewers (MV and OE). During the second stage the same two reviewers, blinded to each other’s decisions, assessed the full texts of potentially eligible studies. Reasons for exclusion at the full-text stage were documented. Any disagreements between the reviewers at any stage were resolved through discussion and consensus. The search and selection process results are presented using a PRISMA-ScR flow diagram in the third stage^25^ (Figure 1).

### Data extraction and synthesis

**FIGURE 2:**
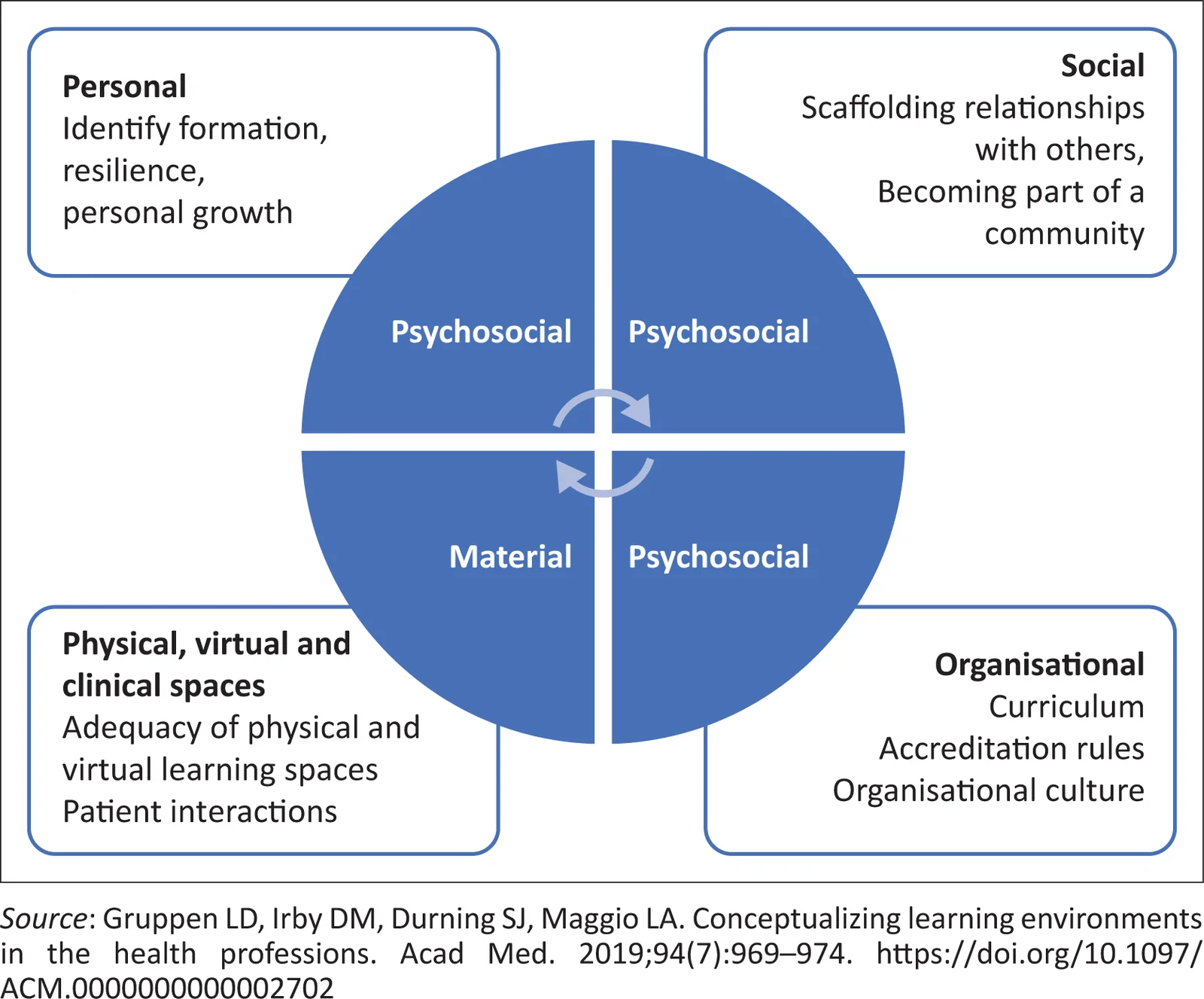
Components of the clinical learning environment.

A data extraction tool was developed by the researchers (Online Appendix 2). This tool systematically charts key attributes, including the title, author, year of publication, journal, country of publication, type of study, study purpose, sample size, presence of an evaluation, evaluation tools used, enablers, obstacles, strategies for improvement, and the advantages of primary care as a CLE. Two reviewers conducted the data extraction, which was subsequently reviewed and discussed with the research team to ensure accuracy and consistency. Data charting and initial coding were conducted by a single researcher using a piloted extraction form, based on the Aksey and O’Malley framework.^24^ A hybrid thematic approach was applied: preliminary codes were informed by the Gruppen framework (Figure 2), while additional codes were generated inductively as new concepts emerged from the data. Related codes were compared and grouped into broader thematic categories based on the principles of reflexive thematic analysis.^26^ This process involved repeated review and refinement of the coding structure with the research team. The coding structure and thematic synthesis were discussed with the research team to minimise individual interpretive bias. Disagreements regarding coding or thematic allocation were resolved through discussion until consensus was reached as recommended by health professions education research methodology.^27^

### Ethical considerations

Ethical clearance to conduct this study was obtained from the University of Pretoria Faculty of Health Sciences Research Ethics Committee (No. 546/2024).

## Results

The search of four databases yielded 627 articles. The manual search yielded another 24 articles. After removing 309 duplicates, the remaining 342 articles were selected for review. Two reviewers independently reviewed the titles and abstracts, selecting 147 articles for full-text review. Of these, 143 full-text articles were accessible and subsequently reviewed by two reviewers via the PCC framework (Table 1), which applied the predefined inclusion and exclusion criteria. This process resulted in the inclusion of 34 articles in the review.

### Study characteristics of the 34 included studies

The selected articles were geographically diverse. Eleven studies were conducted in the United Kingdom^3,28,29,30,31,32,33,34,35,36,37^: three each in the Netherlands,^38,39,40^ Sweden,^41,42,43^ Australia^44,45,46^ and South Africa^14,47,48^; two each in New Zealand,^49,50^ Indonesia^51,52,53^ and the United States [US]^53,54^; one each in Turkey,^55^ Canada,^56^ Ireland^57^ and Denmark^58^; and a multicountry survey was conducted in 10 African countries.^59^ More than half of the studies were conducted in Europe (56%), and only one-fifth (20%) were conducted in lower and middle-income countries: South Africa, Turkey, Indonesia, and the multicountry African study. Only 6% of the studies were conducted in the United States. Regarding study design, 13 were qualitative, 10 were cross-sectional, and 8 were mixed-methods, one each was a workshop, nominal group technique and a development of a psychometric tool. Among the 34 included studies, most investigated the experiences or perspectives of medical students (*n* = 28). Only one study focused on patients,^45^ two examined both patients and students,^29,41^ two explored faculty perspectives,^14,47^ and one investigated clinical teachers.^50^ The enablers and obstacles of the CLE are reported in a table based on a modification of the framework of Gruppen et al. The original framework comprised four interlinked components: personal, social, organisational and space (Figure 2). In the modification, a fifth category, the ‘mentor or teacher’, was added, which intersects with the other categories. The ‘spaces’ category was also clarified to include the clinical interaction with patients in the physical space. Recurrent themes were identified, consolidated, and coded, yielding 31 distinct enablers and obstacles (Table 2). Frequency refers to the number of included studies that identified a specific theme, counted once per study.

**TABLE 2:** Enablers and obstacles to a conducive clinical learning environment.

Domain	Enabler or Obstacle	Description	*n*
Mentor	Enabler	Acts as a skilled, enthusiastic mentor who models professional behaviour^3,28,32,36,37,42,51,52,55,56^	11
	Enabler	Facilitates student autonomy through graded responsibility^3,56^	2
	Enabler	Provides timely and constructive feedback to support learning^29,40^	2
	Obstacle	Teachers lack institutional support, and clear career pathways^14,47^	2
	Obstacle	Authoritarian or controlling teaching style^55^	1
Organisational	Enabler	Longitudinal rotations that provide continuity of learning^14,36^	2
	Enabler	Alignment between university objectives and CLE^14^	1
	Enabler	Quality feedback processes embedded in the programme^28,31,37^	3
	Obstacle	Courses at university are overly theoretical with insufficient clinical application^55^	1
	Obstacle	A lack of structured staff development for teaching^14^	1
	Obstacle	Discordance between what is taught at university and what is modelled in practice^38^	1
	Obstacle	Poor communication between the university and the clinical learning site^42^	1
Physical, virtual, and clinical space	Enabler	Manageable patient load allowing for adequate student learning time^30,33,39^	3
	Enabler	A variety of patient conditions are encountered^36^	1
	Enabler	Dedicated consulting space for students^40^	1
	Enabler	Approachable and willing patients who engage with students^37,41^	2
	Enabler	High-quality care is provided in the clinical setting^41^	1
	Enabler	Availability and creative use of ICT to enhance learning^14,34^	2
	Obstacle	Travel distance and associated costs lead to fatigue and loss of clinical time^3,14,37^	3
	Obstacle	Inadequate space for consultations^54^	1
	Obstacle	Inadequate internet connectivity^59^	1
Personal	Enabler	High levels of student motivation^52^	1
	Enabler	Independence in clinical work	1
	Enabler	Autonomy in decision-making^3,40,42^	3
	Obstacle	A lack of student agency^5^	1
	Obstacle	Fatigue and tiredness affect performance^55^	1
	Obstacle	Feelings of insecurity in clinical settings^41,53^	2
Social	Enabler	An atmosphere that is warm, welcoming, and relaxed^3,34,40,55,57^	5
	Enabler	Positive relationships with teachers and staff^40,42,52^	3
	Enabler	Being part of the clinical team^3,34^	2
	Obstacle	A lack of social opportunities in placement^37^	1
	Obstacle	Hierarchical culture limiting student engagement^29,55^	2

Note: Please see the full reference list of Eales O, Brits H, Lubbe I, Moyo-Chilufya M, Van Rooyen M. A conducive clinical learning environment in primary care as a wicked problem: A scoping review of enablers, obstacles, and strategies. Afr J Prm Health Care Fam Med. 2026;18(1), a5246. https://doi.org/10.4102/phcfm.v18i1.5246

CLE, clinical learning environment.

### Enablers and obstacles

**FIGURE 3:**
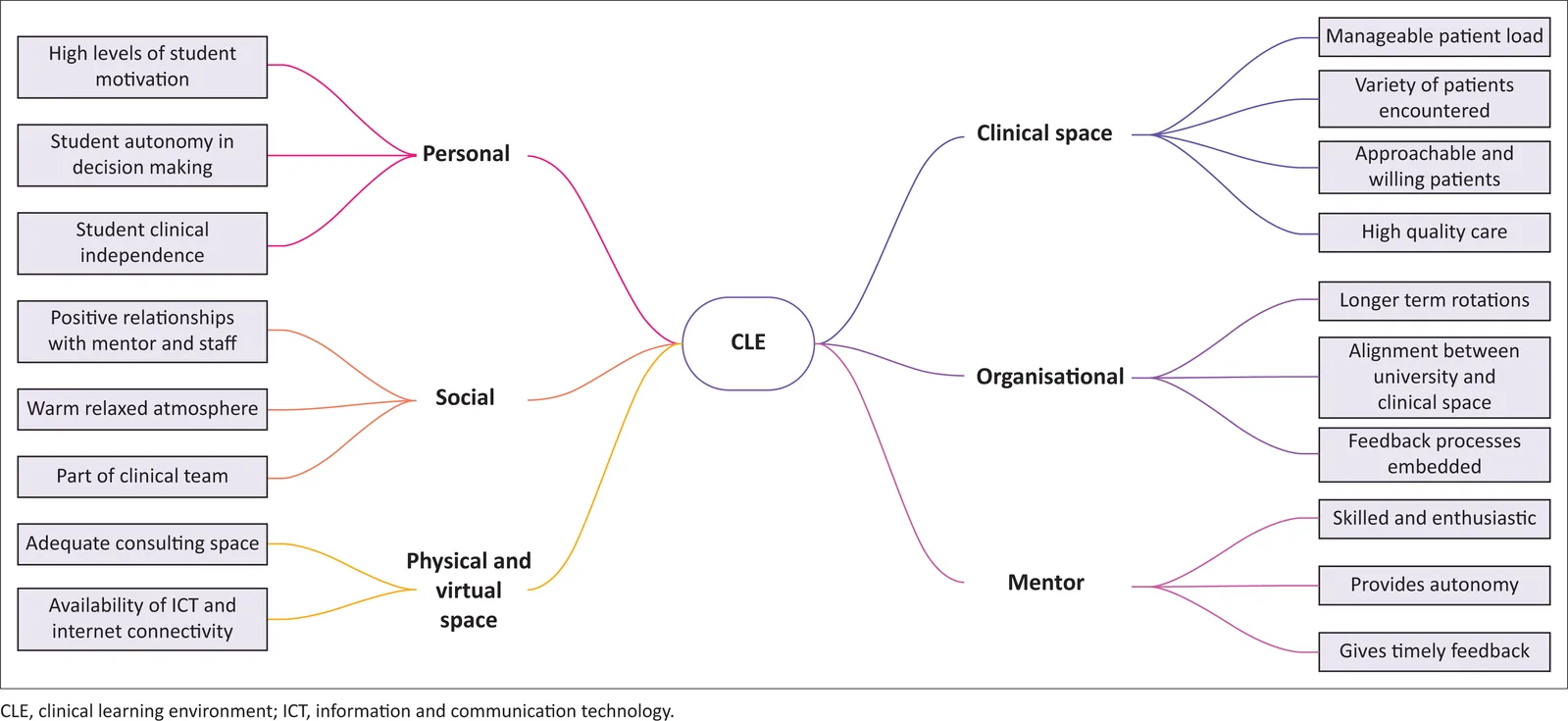
The enablers of a conducive clinical learning environment.

**FIGURE 4:**
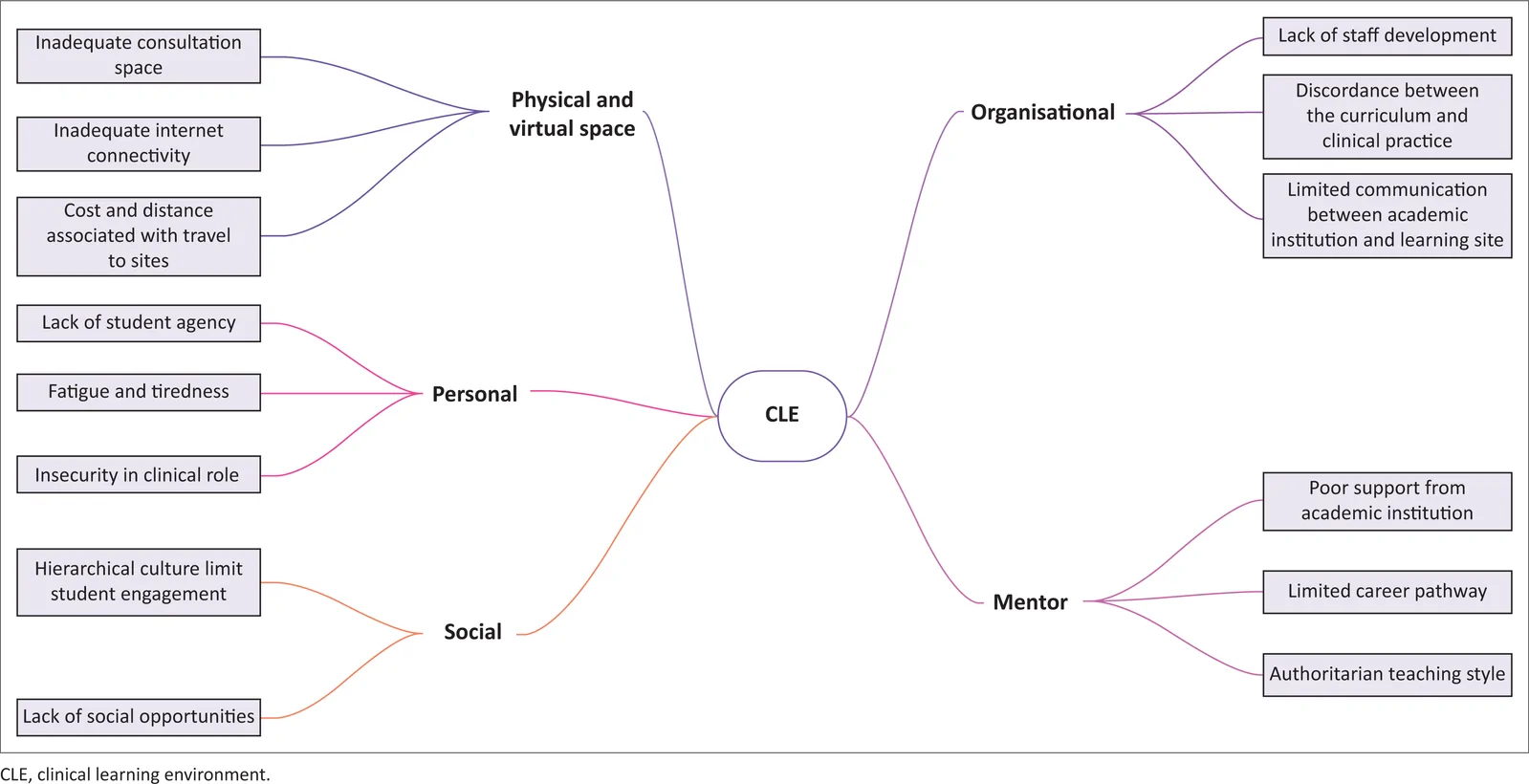
The obstacles to a conducive clinical learning environment.

The category most frequently reported was mentor or teacher (*n* = 18), with common enablers including skilled and enthusiastic mentors who model professionalism, facilitation of student autonomy, and providing constructive feedback. Obstacles in this category included a lack of institutional support, authoritarian teaching styles, and poor mentor-university communication. Spaces (physical, virtual, and clinical) (*n* = 14) featured enablers such as a manageable patient load, variety of clinical conditions encountered, dedicated student space, and engaged patients. Obstacles included long travel distances, inadequate consultation space, and poor internet connectivity. The social domain (*n* = 13) was characterised by enablers such as a warm, welcoming atmosphere, positive relationships with staff, and integration into the clinical team. Obstacles included a lack of social opportunities and hierarchical cultures. In the organisational category (*n* = 10), enablers included longitudinal rotations, institutional and clinical learning goal alignment, and embedded feedback processes. Common obstacles included overly theoretical courses, insufficient staff development, misalignment between taught and modelled practice, and poor communication with clinical sites. Finally, the personal category (*n* = 9) included enablers such as motivation, independence and decision-making autonomy, while obstacles encompassed a lack of agency, fatigue, and insecurity in clinical contexts. The enablers and obstacles are summarised thematically in Figure 3 and Figure 4.

### Strategies to improve the clinical learning environment

Only six studies explicitly described strategies to enhance the CLE.^14,38,45,48,49,55^ Reported strategies included implementing structured faculty development programmes^54^; providing students with training to improve their sense of agency^48^; involving non-faculty stakeholders in curriculum design^50^; strengthening alignment between universities, clinical sites, and departments of health^14^; and fostering collaboration with patients in the planning and delivery of student training.^45^

## Discussion

The development of a competent physician is a complex endeavour that includes multiple competencies such as medical expert, collaborator, advocate and communicator. The role of a conducive CLE in the development of many of these competencies are clear from this review, such as the role of mentor and/or clinical teacher in student development and the role of relationships to develop collaboration and communication.^48^

### Mentor and/or clinical teacher

This was the domain most often mentioned in this review, and it intersected with all the other domains. The mentor’s influence extended beyond immediate supervision to shaping the culture of the learning environment and supporting students’ professional growth. The importance of the clinical mentor is a universal theme across different types of CLEs, from primary care to academic hospitals.^8,21^ It seems the difference is that in an academic teaching hospital, mentorship is provided through multiple teachers. In contrast, there is more continuity and attention from a smaller group of mentors in primary care.^8,35,38^ An enthusiastic and skilled mentor can facilitate learning even in suboptimal conditions.

### Autonomy and agency

The concept of appropriate student autonomy or freedom also cuts across multiple domains. Autonomy is closely related to agency, defined as students’ ability to make decisions and take ownership of learning.^56^ Autonomy appears to be equally important in the hospital and primary care environments. Autonomy was facilitated by adequate space for independent consultations, graded responsibility and mentor support.^38,42^ However, obstacles such as controlling teaching styles or a lack of student agency hindered the benefits of autonomy.^50,55^ Supporting both autonomy and agency requires explicit preparation of students and staff.

### Relationships

Positive relationships across all domains of the Gruppen framework were central to shaping the CLE, fostering belonging, engagement, and professional growth. From peers and mentors to patients and institutions, the quality of these interactions strongly influenced students’ well-being, motivation, and confidence. The importance of relationships in the CLE is echoed in a scoping review by Hepburn et al.^21^ on rural and remote clinical placements. It appears that in primary care and rural and remote placements, the relationships with staff, peers, and patients are more critical than in hospital-based CLEs.^8^ Despite their importance, little curricular attention is devoted to teamwork and relationship-building skills within CLEs.^8^

### Logistics

The challenge of the availability of appropriate space and ICT infrastructure for students in the CLE is not unique to primary care, as this is also shared with academic hospitals.^60^ A unique challenge to the primary care platform is the cost and time of transport to the CLE.^4,15,19^ These logistical obstacles directly affect equity of access, with students from less resourced backgrounds being disproportionately disadvantaged.^61^ Extended travel time can also lead to fatigue and loss of clinical time.

### Strategies to improve the clinical learning environment

The strategies identified in this review primarily targeted organisational domains, reflecting the institutional perspective of most researchers.^62^ These included structured faculty development, fostering student agency, aligning universities and clinical sites, and engaging patients as partners. However, limited attention was given to systemic obstacles such as transport and ICT infrastructure.

### A wicked problem

The complexity and wide-ranging findings of this review suggest that designing and sustaining a conducive CLE in primary care can be understood as a wicked problem.^16,17,18^ The 10 characteristics of wicked problems are mapped against the results of the scoping review in Table 3.

**TABLE 3:** The clinical learning environment as a wicked problem.

Characteristics of wicked problems	Application to scoping review
1. No Definitive Formulation.	A conducive CLE is formed by the interaction of multiple factors and role-players, making it difficult to define.^3,38,59^
2. No Stopping Rules: The problem is never truly ‘solved’.	Factors such as staff development and communication between universities and clinical sites are ongoing.^14,52^
3. Solutions are False or Not True: Solutions are rated as ‘better or worse’.	Educational strategies such as longitudinal placements improve learning but vary in effectiveness depending on context.^14,37^
4. No Immediate or Ultimate Test: You cannot instantly test if a solution works; effects can only be known long-term.	The outcomes of interventions such as improving alignment between universities and departments of health, only become apparent over time.^14^
5. One-Shot Operation: Every attempt counts, as you cannot easily undo actions or use trial-and-error.	Solutions such as staff development and infrastructure improvement are long-term investments.^41,55^
6. No Exhaustive Solution Set: There is no definitive list of potential solutions.	Multiple solutions are suggested, such as mentorship development, curriculum redesign and use of ICT.^14,34^
7. Essentially Unique: No two wicked problems are exactly alike, meaning there are no pre-existing, off-the-shelf solutions.	Learning environments differ in terms of infrastructure, staffing, resources and patient loads.^30,33,48,59^
8. Symptom of Another Problem: A wicked problem is often a symptom of a deeper, underlying issue.	The struggle for a conducive CLE reflects broader health system and organisational problems and constraints.^28,42,46,54^
9. Multiple Explanations: There are many ways to explain the causes, and the chosen explanation dictates the solution.	Multiple stakeholders such as the health system, universities and students have conflicting interests and perspectives.^3,32,34,52,56^
10. No Right to Be Wrong: the consequences can have significant, long-term impacts.	Inadequate training and supervision of students can have significant impact on the health system and patient safety.^3,47^

Note: Please see the full reference list of the article Eales O, Brits H, Lubbe I, Moyo-Chilufya M, Van Rooyen M. A conducive clinical learning environment in primary care as a wicked problem: A scoping review of enablers, obstacles, and strategies. Afr J Prm Health Care Fam Med. 2026;18(1), a5246. https://doi.org/10.4102/phcfm.v18i1.5246, for more information.

CLE, clinical learning environment.

Although none of the studies referred to a conducive CLE directly as a wicked problem, all 10 characteristics of wicked problems can be mapped from the results. In the CLE, mentorship, autonomy, relationships, organisational processes, and logistics are tightly interwoven. Interventions that address one aspect, such as faculty development, may not succeed if other obstacles, such as transport or space constraints, remain unresolved. Similarly, strategies effective in one context (e.g. longitudinal placements in the UK) may be impractical in low-resource settings. Given the complex and context-specific nature of the CLE, the strengths and limitations of this study should be considered in the application of these findings.

### Strengths and limitations

This study followed a systematic and rigorous approach that included pre-registration to improve transparency. Major biomedical, educational, and transdisciplinary databases were searched, leading to a significant number of studies (*n* = 627). Multidisciplinary authorship further strengthened the study, with the lead author’s primary care experience providing contextual insight. The exclusion of grey literature was a limitation that potentially omitted implementation-focused evidence and introduced publication bias. The role of the lead author as coordinator for medical students during their primary care rotation may have introduced interpretive bias, although mitigated through team-based discussions.

### Educational implications for primary care

**FIGURE 5:**
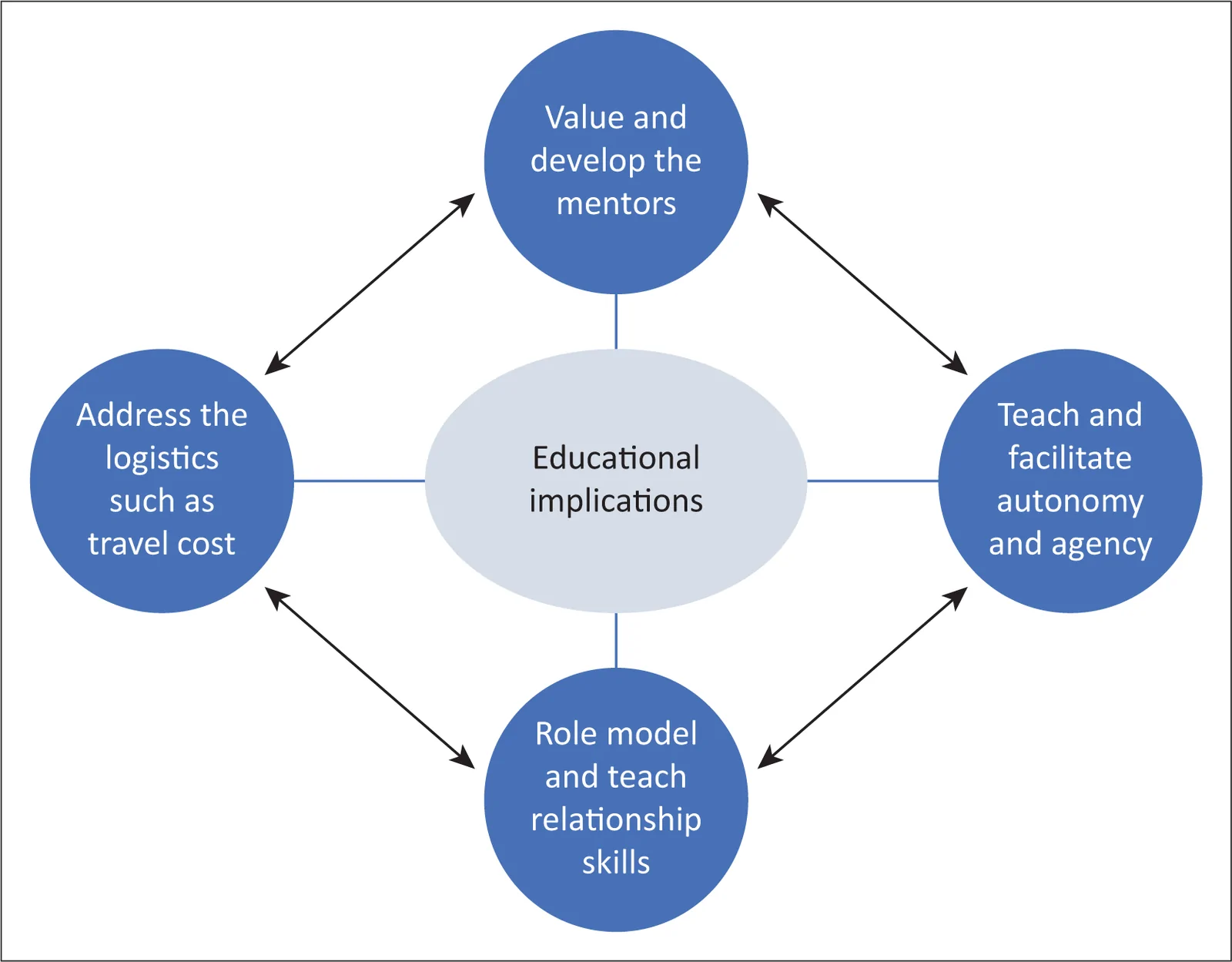
Educational implications.

The following implications cannot be viewed or addressed in isolation but should be seen holistically and addressed concurrently (Figure 5).

### Mentor and/or Clinical teacher

Training institutions should formally acknowledge the central role of mentors and/or clinical teachers and invest in sustained faculty development that integrates clinical expertise with educational skills.^14^ This support should extend to mentors and/or clinical teachers not directly affiliated with universities, ensuring their inclusion in curriculum planning and assessment.^50^ They should be supported to pursue further professional development through short courses, diplomas or postgraduate courses, with protected time allocated for their teaching activities.^36,46,50^ Mentors should be developed to facilitate these processes in order to shift the focus from what students are taught, to how students learn.^14,48^ Mentors should be equipped with the principles of adult learning, and shift the focus away from what students are taught to how students learn.^14,48^

### Autonomy and agency

Curricula should incorporate a structured approach to autonomy and agency, gradually preparing students to assume responsibility for patient care while equipping mentors to facilitate this process.^3,48^ Training should explicitly distinguish autonomy and agency, highlighting students’ active role in shaping their own learning.^39,42^ Students should be guided to actively set personal learning goals and to reflect on the learning process.

### Relationships

Relationship-building and teamwork must be recognised as core student competencies. Explicit teaching of interprofessional collaboration, conflict resolution, and patient partnership should be integrated into primary care placements.^3,34,40^ Patients should be engaged as active contributors to student learning and their feedback on student interactions should be sought and valued.^37,41^

### Logistics

Finally, logistical obstacles must be addressed systematically, particularly transportation, space, and Information and Communication Technology (ICT) infrastructure.^3,14,37^ Universities and health systems should collaborate to ensure equitable access, including subsidised travel, adequate consultation space, and reliable digital connectivity.^14,42^ This should include investment by universities, such as refurbishment of consultation rooms and internet access for students.^34,40^ For cooperation and investment to be sustainable, long-term service level agreements need to be developed between stakeholders.^14^

## Conclusion

A conducive CLE in primary care is central to the development of clinician competence. Relationship-building skills and logistics appear especially important enablers in the primary care environment because of its distributed and variable nature. However, given the complex and ‘wicked’ nature of the CLE, improvement strategies should not be pursued in isolation but through coordinated, multilevel approaches involving universities, clinical sites, health authorities, students and communities. Recognising and addressing this complexity may be key to developing competent future clinicians.
